# Waiting-list interventions for children and young people using child and adolescent mental health services: a systematic review

**DOI:** 10.1136/bmjment-2023-300844

**Published:** 2024-02-01

**Authors:** Althea Z Valentine, Sophie S Hall, Kapil Sayal, Charlotte L Hall

**Affiliations:** 1 NIHR MindTech MedTech Co-operative, Institute of Mental Health, Mental Health and Clinical Neurosciences, University of Nottingham, Nottingham, UK; 2 University of Nottingham, Nottingham Clinical Trials Unit, Nottingham, UK; 3 Institute of Mental Health, School of Medicine, Mental Health and Clinical Neurosciences, University of Nottingham, Nottingham, UK; 4 NIHR Nottingham Biomedical Research Centre, Institute of Mental Health, University of Nottingham, Nottingham, UK, University of Nottingham, Nottingham, UK

**Keywords:** child & adolescent psychiatry

## Abstract

**Question:**

Children and young people experience delays in assessment and/or treatment within mental health services. The objective of this systematic review, funded by the Emerging Minds Network, was to explore the current evidence base for mental health waiting list interventions to support children and young people.

**Study selection and analysis:**

A literature search was conducted in MEDLINE, PsycINFO, Web of Science and the Cochrane databases from 2000 to 2023 (last searched October 2023). Included studies described interventions to support children and young people and/or their family while on a waiting list for child and adolescent mental health services. Titles and abstracts were screened independently by two reviewers, data were extracted by one reviewer, confirmed by a second and a narrative synthesis was provided.

**Findings:**

Eighteen studies including 1253 children and young people were identified. Studies described waiting list interventions for autism spectrum disorders, eating disorders, generic conditions, transgender health, anxiety/depression, self-harm and suicide and behavioural issues. Many interventions were multicomponent; 94% involved psychoeducation, other components included parental support, bibliotherapy and coaching. Duration of the interventions ranged from a single session to over a year; 66% involved face-to-face contact. All studies demonstrated benefits in terms of improved clinical outcomes and/or feasibility/acceptability. Evidence for service outcomes/efficiency was largely unexplored. Limitations of the underpinning research, such as sample size and low-quality papers, limit the findings.

**Conclusions:**

There is limited research exploring waiting list interventions, however, the findings from small-scale studies are promising. Further research using robust study designs and real-world implementation studies are warranted.

**WHAT IS ALREADY KNOWN ON THIS TOPIC**

- Waiting lists for child and adolescent mental health services are growing worldwide, therefore there is a need to understand how referred patients on waiting lists can be better supported.

**WHAT THIS STUDY ADDS**

- Waiting list interventions have the potential to support children, young people and their families while waiting for mental health assessment and/or treatment.
- Psychoeducation lends itself to being offered as a waiting list intervention.

**HOW THIS STUDY MIGHT AFFECT RESEARCH, PRACTICE OR POLICY**

- Waiting list interventions show promise in supporting children and young people accessing child and adolescent mental health services; further research is warranted to further understand the benefits to the service, the patient and how best to develop and implement these in practice.

## Background

Children, young people and their families report long waiting lists for assessment, diagnosis and treatment once referred to child and adolescent mental health services (CAMHS) in the UK. A survey of almost 14 000 young people found that 44% waited more than a month for mental health support, 26% of which have attempted to take their own life while waiting for support.^1^ Probable mental health disorders in those aged 17–19 years increased from 1 in 10 (10.1%) in 2017 to 1 in 4 (25.7%) in 2022. In those aged 7–16 years, there was an increase from one in nine (12.1%) in 2017 to one in six (16.7%) in 2020, although rates have since remained more stable.^2^ This is reflected in referrals to CAMHS, where a 109% increase in referrals has been seen between April 2019 (31 720 referrals) and April 2022 (66 389 referrals).^3^ Waiting list times may influence engagement with services, deterring families from seeking help^4^ and negatively impacting engagement with therapy.^5–7^ An exacerbation of mental and physical health issues may also be experienced while on waiting lists.^7^ The James Lind Alliance (JLA) identified which interventions are effective in supporting children and young people on waiting lists as a top-10 priority (https://www.jla.nihr.ac.uk/priority-setting-partnerships/Mental-health-in-children-and-young-people/top-10-priorities.htm).

Over the past 20 years, there has been an increasing body of research on waiting list strategies within mental health services. It has been recommended that interim interventions (such as bibliotherapy and relaxation training) should be considered for patients facing long waiting times, as well as improving the administrative management of waiting lists, for example, using clear eligibility criteria, screening and opting in.^8^ Research has focused mainly on service initiatives to manage lists rather than interim patient-facing interventions. A recent systematic review identified 20 articles, including studies of mental health services for children and young people (n=10), family or adult (n=4), primary care (n=3) and university counselling (n=3). The paper detailed strategies services have used to manage waiting list demands including demand management approaches such as walk-in models, triage, multidisciplinary care, patient-led approaches and service delivery changes.^9^

To improve service delivery and better support children and young people on waiting lists, there is a need to understand what waiting list interventions are currently offered and the evidence base to support their use; we conducted a systematic review to explore this.

## Objective

The objectives of the review were to: (a) explore, summarise and assess the quality of the available peer-reviewed evidence-base for interventions for children and young people on waiting lists for mental health services; (b) provide an overview of the characteristics, nature and diversity of the interventions and (c) explore the evidence in terms of outcomes of interest including clinical outcomes, service efficiencies and user impact, acceptability and reported barriers and facilitators to engagement. In doing so, we identify gaps, deficiencies and trends in the current evidence to help underpin and inform future research.

## Methods

### Study selection and analysis

The objectives, inclusion criteria and methods for this review were specified in advance (table 1), following appropriate guidelines^10^ (see online supplemental file 1 for the protocol). Relevant terms were noted following a limited database search, from which a comprehensive list was created by clinical and academic experts. The main search was run in three databases MEDLINE (Ovid), PsycINFO (Ovid) and Web of Science, restricted by date from 2000 to 2023 to ensure interventions were relevant, and by those published in English. The search was initially undertaken on 10 September 2021 and updated October 2023. A search was also run in Cochrane Trials in September 2021 only. Free-text search terms related to waiting (eg, “wait*”, wait* adj5 initiative, wait* intervention, wait* adj time, Wait* adj5 length, Wait* adj5 duration, Access adj5 delay, Wait* adj5 access) and children and young people mental health (eg, psych*, behav*, CAMHS, Child*, “mental health” or depress*). Subject/Medical Subject Headings related to mental health (eg, Mental Health/Services/ Disorders, Pediatrics, Child and Adolescent Psychiatry/Psychology/Psychotherapy/Psychopathology). Following data extraction, backward and forward citation chasing was conducted using a citationchaser^11^ (https://estech.shinyapps.io/citationchaser/) looking for all records citing (forward citation chasing) or referenced (backward citation chasing) in one or more of the included articles, this search was updated October 2023.

10.1136/bmjment-2023-300844.supp1 Supplementary data

**Table 1:** Inclusion and exclusion criteria

PICO criteria	Inclusion criteria	Exclusion criteria
Population	Children and young people (up to 18 years) referred to a specialist mental health service for assessment or treatment of any mental health disorder. Participants may also be the families/carers or healthcare providers of these participants.	No exclusion criteria.
Intervention	We defined ‘waiting list interventions’, as an intervention used to support participants and/or their family while on a waiting list for mental health assessment, diagnosis and/or treatment. There were no restrictions on the frequency, timing, geographical location or healthcare setting, or those administering the intervention, but these details were noted in the data extraction as important features.	The following were excluded: waiting list management from a service perspective, models for healthcare delivery, appointment scheduling or improving access, opinions on general service satisfaction or clinical changes not linked to waiting list interventions, interventions that could be a waiting list intervention but are not tested as such, preventative trials not at the point of referral.
Comparators	The intervention must have been used to support participants while on a waiting list for assessment or treatment in a mental health service; any comparators were considered.	No exclusion criteria.
Outcomes	Outcomes included clinical outcomes, service efficiencies and user impact, acceptability and reported barriers and facilitators to engagement.	No exclusion criteria.
Types of sources of evidence	Evidence sources included qualitative and quantitative research studies and conference abstracts that reported clinical, cost-effectiveness and/or perceptions (feasibility/usability). There were no restrictions on the study design.	Non-peer-review articles and study protocols were excluded.

**Figure 1:**
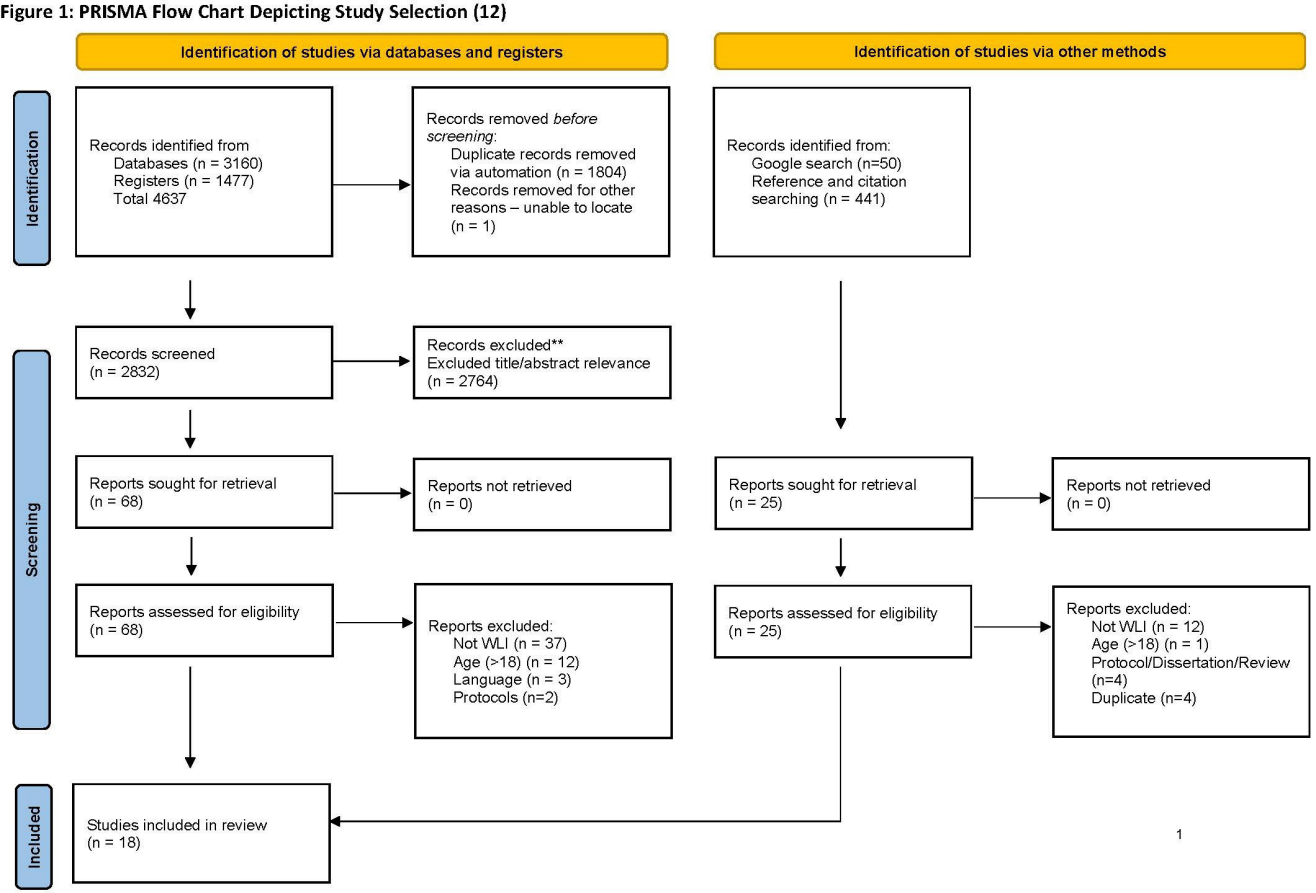
Flow diagram showing the selection of reports included in the review. WLI, waiting list intervention.

The retrieval method was the same across all search methods. All identified citations were collated and uploaded into EndNote V.x9.3.3 (Clarivate Analytics, Pennsylvania, USA) and 1804 duplicates removed using Endnote automated tools. Following pilot testing the study selection, all titles and abstracts were screened independently against the inclusion criteria in Endnote (AZV) and following data importation to Excel (CLH/SSH). Two researchers independently reviewed the full texts of selected articles and the reason for exclusion was noted. Any disagreements that arose were resolved through discussion. The results of the search are presented in a Preferred Reporting Items for Systematic Reviews and Meta-Analyses (PRISMA) flow diagram^12^ (see figure 1).

Data were extracted using a standardised form by one researcher (AZV) and verified for accuracy by one of two reviewers (CLH and SSH), discrepancies were resolved via discussion. The first three papers were used to pilot the data extraction tool, minor modifications were made to ensure that relevant data were collected (eg, including the percentage of eligible participants recruited). Extracted data included study and population characteristics, descriptions of study aims and intervention and barriers and facilitators. Outcomes of interest were clinical (any measure and timepoint), service efficiency (eg, reduction in waiting times, intervention costs, etc) and user impact (feasibility and acceptability). Missing data were recorded and authors were not contacted for additional information. Because of the heterogeneity of the included studies, a narrative synthesis was used.

A rapid appraisal of the level of evidence was assessed using the Oxford Centre for Evidence-Based Medicine (OCEBM; https://www.cebm.ox.ac.uk/resources/levels-of-evidence/ocebm-levels-of-evidence). The levels of evidence ranged from 1 to 5, where 1 was the highest quality. One reviewer (CLH) conducted the appraisal, which was verified by a second reviewer (SSH), any disagreements were resolved via discussion.

## Findings

A total of 5078 studies were identified, imported into EXCEL and full texts read for relevance (n=93), if not relevant the reason for exclusion was noted (see figure 1), 18 papers were identified as meeting the eligibility criteria.^13–30^ The characteristics of the 18 included studies are presented in online supplemental table 2 and online supplemental table A1.

10.1136/bmjment-2023-300844.supp3 Supplementary data

10.1136/bmjment-2023-300844.supp2 Supplementary data

### Quality

The quality appraisal revealed most studies were of low quality. Although there were four randomised controlled trials (RCTs), only one met the prespecified power sample size and was given a OCEBM level 2 quality rating.^22^ However, the sample size was small (n=60) and there was insufficient information on how the power calculation was performed.^22^ This study along with another small study including 51 parents/caregivers and 36 young people^24^ were designed to assess effectiveness. The remaining RCTs were designed to assess feasibility/acceptability, and thus were not fully powered^14 21^; these were downgraded from possible 2 to 3 ratings. We noted the importance of these studies to inform the future development of efficacy RCTs. From the remaining papers, all studies were classified as OCEBM level 3–5, most described an evaluation of implementation within a single service, comparing scores at baseline and postintervention. Typically, the studies did not have a comparator group.

### Population

All participants were on waiting lists for mental health services delivered in healthcare settings relevant to the country of the study. Five were conducted in the USA, four in Australia, four in the UK, three in Canada and two in Ireland. The reasons for being on a waiting list included: autism spectrum disorders (ASD; n=5), eating disorders (n=4), generic CAMHS referrals (n=3), transgender health (n=2), anxiety/depression (n=2), self-harm/suicide (n=1) and behavioural issues (n=1). All ages of children were included in the review from under 5 to 18 years. Those under 5 were all referred to CAMHS for ASD or generic reasons. Where reported, all studies included a mix of genders, with ASD and behavioural issues involving predominantly male participants and eating disorders, anxiety and depression, predominantly female participants. Most studies were small with 7 studies involving fewer than 20 participants and 7 studies between 21 and 100 participants. The larger studies included one for 125 families of adolescents with self-harm or suicidality behaviours, one for 268 families on eating disorders waiting lists and the two transgender health studies including 142 and 194 participants, respectively. Recruitment was an issue across multiple studies and small sample size was frequently mentioned as a limitation of the research.

### Intervention type and duration

The interventions varied considerably and most of the interventions were multicomponent. All but one^14^ of the interventions involved at least some psychoeducation aspects, many involved providing parental support (n=11) and/or provided a self-help manual, video or other reading material for parents/young people (n=10). Coaching (n=3) and referrals or direction to additional support (n=2) were also used. Most interventions (n=12) involved some face-to-face contact and were aimed at parents alone (n=11) or parents alongside children and young people (n=5). Only one study^15^ gave children and young people the option to complete the intervention with or without their parent/carer (depending on their preference). The duration varied from a single session to >12 sessions. Several studies were a single-session intervention with follow-up support provided where required.

### Aims of reviewed studies

All 18 papers aimed to evaluate the waiting list intervention. In seven, this was generally in terms of effectiveness and parental satisfaction. Five papers aimed to assess feasibility and acceptability of the interventions. The remaining papers were to explore the impact on mental health, to document the creation and implementation of a waiting list intervention, to improve parental knowledge and self-efficacy, to support families and to reduce the waiting list.

### Methodology adopted

The research design varied considerably with two RCTs and two randomised controlled feasibility studies, a further five feasibility studies were conducted which were generally quasi-experimental or mixed methods, other research designs included service evaluations, action research and qualitative studies.

### Key findings (evidence established)

#### Clinical outcomes

The clinical outcomes varied considerably across studies, the main clinical outcomes were changes in child symptoms or behaviours and parental anxiety, depression and stress. Further details about the outcomes in individual studies are presented in the online supplemental file 1. Overall, clinical outcomes were limited in terms of the small sample sizes.

#### Child-related outcomes

The Strengths and Difficulties Questionnaire (SDQ) was used in two studies.^13 22^ One study found significant reductions on the conduct, emotional and hyperactivity subscales which demonstrated a positive effect of the intervention.^13^ The other study showed a significant improvement on the mean total SDQ score between baseline and 6-month follow-up.^24^ Most studies used parent-reported measures for outcomes relating to the child, only four studies used child or young person self-report measures, or included both parental and child self-report measures.^15 18 21 24^

Behavioural changes were assessed in three of the four eating disorders studies in terms of child weight and eating disorder symptoms. A positive impact was noted in two studies,^17 26^ with significant weight gains reported in both studies; one study^26^ additionally noted a decrease in eating disorder behaviours. No impact on eating disorder symptoms was found in another.^24^

#### Parent-related outcomes

There were contradictory findings on whether the waiting list interventions improved parental stress^14 23^ and parental depression and anxiety.^15 18 21 27 29^ Improvements were noted in quality of life and parent self-efficacy,^17^ as well as a reduction in the proportion of families with unhealthy family functioning.^18^

#### Service efficiencies

Three service efficiencies were reported. McGarry *et al* found that when a brief consultation and advice appointment was provided as a waiting list intervention, fewer families dropped out compared with those who received treatment as usual.^22^ In a service report of three different methods of family-based treatment for eating disorders, the authors suggested that there was a reduced burden on staff and increased efficiency of appointments, potentially reducing waiting lists, however, no data were given to support this.^28^ The service evaluation of the First Assessment Single-Session Triage intervention for transgender children and young people found a reduction in wait time from 14 to 4 months^19^ after implementing the intervention. Costs were mentioned in four papers, but these were not related to the economic assessment of the intervention. Two studies reported that the intervention was cost-neutral to participants but did not discuss how they determined this,^14 23^ one study reported that it was a low-cost intervention^26^ and another that they provided play materials^20^ but none provided details on costs or information about cost savings that would be useful in terms of monitoring service efficiencies or intervention costs.

#### User impact

In terms of user impact, there were high ratings in terms of parental acceptability, satisfaction and/or feasibility.^13–15 20–22 27 28 30^ Parents reported increased understanding and/or greater self-efficacy^16 23^ as well as feeling empowered.^24 28^ In a pilot study using a brief solution-focused approach, clinicians reported that they were highly satisfied with the waiting list intervention and enthusiastic about the effectiveness of the pilot.^25^

Limitations of interventions were also noted in terms of enablers and barriers, with some families feeling they would like more frequent and intensive intervention,^23^ an advice line for support, a parental support group and a shorter waiting time.^13^ Within the psychoeducation elements, there was mixed feedback with some families feeling that the information was too generic, and some families did not find the content as helpful as expected.^26 27^ Some children and young people reported difficulties in attending the intervention because it was held during the school day.^21^ Low recruitment rate was noted as a barrier in some studies,^17 21^ with authors postulating that this may be because of parental disempowerment and fear of losing their place on the waiting list or that support may be delayed because of their participation.^26^ However, once participants were recruited, waiting list interventions generally had low drop-out rates.^13 21 22^

## Discussion

This review considered the published literature for waiting list interventions for children and young people and families waiting for mental health assessment and/or treatment. It aimed to (a) explore, summarise and assess the quality of the available peer-reviewed evidence base for interventions for children and young people on waiting lists for mental health services; (b) provide an overview of the characteristics, nature and diversity of the interventions and (c) explore the evidence in terms of outcomes of interest including clinical outcomes, service efficiencies and user impact, acceptability and reported barriers and facilitators to engagement.

### Evidence base

This review found limited evidence on waiting list interventions; only 18 papers were identified, and the quality of study design was often weak/moderate and often exploratory in nature. Sample size was a common issue and recruitment problems were frequently reported as barriers to engagement. Nevertheless, these papers indicate the potential to use the waiting list period to offer an active intervention.

### Characteristics, nature and diversity of the interventions

Existing waiting list interventions largely fall within recommendations made in previous research, such as bibliotherapy and relaxation training,^8^ alongside coaching interventions for families living with ASD. The majority of papers in the review used psychoeducation approaches. Most waiting list interventions were brief (five or fewer sessions), indicating single-session interventions as a possible avenue for further exploration. Future research may consider whether a stepped-care approach, whereby psychoeducation is offered to all children and young people and/or families on waiting lists, reduces waiting times and subsequent length of treatment. Most waiting list interventions were aimed at the parent, future child-led research may be beneficial, particularly for conditions such as eating disorders and transgender health support which tended to include older adolescents.

### Outcomes of interest

User feedback in general suggests high levels of satisfaction. Parents appreciated receiving support while on waiting lists, with some evidence of lower attrition to the targeted intervention. Given the exploratory nature of many of the studies, few studies reported on clinical outcomes, and these presented mixed results. Nevertheless, several interventions had some supporting evidence for their clinical effectiveness.^13 15 17 18 21 22 24 26 27 29^

Most studies included psychoeducation as a waiting list intervention. This is not surprising as psychoeducation is often a key element of many therapies and can be delivered with minimal therapeutic input. A few studies suggested psychoeducation involved minimal costs, but no studies provided detailed cost-analysis. For some families, the psychoeducation provided was sufficient support so that they did not require further input, thus potentially reducing waiting lists for targeted services. However, feedback on psychoeducation varied with some families feeling that the generic information provided in videos or books was not relevant to their specific family circumstances and therefore deemed less useful than more tailored information provided by healthcare practitioners. It is not possible from the few studies evaluated to assess this in detail, but future research could consider the impact of information dissemination via healthcare practitioners and other sources. Most interventions involved some element of face-to-face contact. This may change following the surge in digital technology in response to the global pandemic and changes in healthcare; the very limited evidence in this review suggests that this may be useful for families on waiting lists and help with service availability across geographic locations^20^ as may the telephone.^23^ Outside of waiting list intervention, research has demonstrated that remotely delivered interventions may be both clinically and cost-effective, reducing the need for input from the small pool of highly trained specialist therapists.^31^ However, adverse events can occur, underlining the need for face-to-face support during the waitlist period or adequate remote monitoring of potential adverse events.

The purpose of this paper was to look at the existing evidence for waiting list interventions in CAMHS. As a result, we did not include study protocols. However, given the increasing pressure on CAMHS resources and the need to support families while they are waiting for assessment and treatment, there is likely to be a growing research focus in this area. For example, the Online Parent Training for the Initial Management of ADHD referral trial is still ongoing, however, the findings will be critical to inform on the clinical and cost-effectiveness, as well as acceptability and feasibility, of using an online approach as a waiting list initiative.^32^

### Limitations

The limitations of the underpinning research limit our ability to draw definitive conclusions through this review, particularly as most of the primary studies are small in nature, and feasibility rather than definitive trials. Several studies reported difficulties in terms of recruitment, this could have led to selection bias in that parents/children and young people who participated were more likely to believe that the intervention would work and consequently more likely to benefit.^13 17^ Some studies reported that recruitment was limited by the exploratory nature of the study.^14^ It may be possible that recruitment was difficult because families were concerned that they might restrict their chances of future intervention or lose their place on the waiting list, this issue needs to be explored further and be clear for future research and clinical practice.^26^ If waiting list interventions are to be implemented within services, there will need to be clear communication around the role and purpose of the waiting list interventions to remove this barrier. We recommend that this communication is co-produced with patient and public involvement.

Additionally, the limitations of any review are relevant in that sources of information may be omitted, this is particularly so because of only using three databases, it is possible that searching additional nursing databases would yield further relevant studies. It was somewhat surprising that a limited number of conditions were identified and perhaps not those which were expected, for example, conditions such as ADHD frequently have a long wait to diagnosis but were not identified in this study. Further research using search terms for conditions such as ADHD may be beneficial. Although we included a variety of search terms and used a comprehensive search strategy to identify the most relevant terms, the search was hindered by the vast number of studies including waitlist control groups; a future systematic review should consider revision of search terms to increase sensitivity.

## Conclusions and clinical implications

This review explored and summarised the available peer-reviewed evidence base for interventions for children and young people on waiting lists for mental health services. It was found that waiting list interventions are being used in some mental health services internationally to help support young people. The limited available evidence indicates some promise of clinical effectiveness and positive user impact for waiting list interventions, there was limited evidence of service efficiencies. In the present review, we have found limited published evidence about user impact (acceptability and feasibility), which has largely focused on the views of parents and clinicians. There needs to be more pragmatic trials and/or service evaluations to evaluate the real-world user impact particularly taking into consideration the views of children and young people. Future research should evaluate waiting list interventions using RCTs and/or gather real-world data on benefits to patients and service efficiencies.

## Data Availability

Data created during this research are openly available from the University of Nottingham data repository at https://doi.org/10.17639/nott.7375.
