# Supplementary material for: Waiting-list interventions for children and young people using child and adolescent mental health services: a systematic review
Source: BMJ Ment Health. 2024 Feb 1;27(1):e300844. doi: 10.1136/bmjment-2023-300844 (PMC10836350; doi:10.1136/bmjment-2023-300844)
Supplement: Supplementary data [file bmjment-2023-300844supp001.pdf]

Summary of Protocol Changes

Protocol Title: WAITING FOR ACCESS INTO TREATMENT "WAIT" STUDY: AN EXPLORATION OF CURRENT INTERVENTIONS OFFERED TO CHILDREN AND YOUNG PEOPLE ON CAMHS WAITING LISTS

	Version Number	***Version Date***
**Current Approved Protocol**	***1.0***	***01/09/2021***
**Amended Protocol**	***1.1***	***19/10/2023***

Rationale for Change:

Following feedback from the reviewers, we were advised to consider re-writing the final document as a systematic review rather than scoping review. Changes were therefore made to the protocol to ensure that the review met guidelines for a systematic rather than scoping review.

In summary, the changes included the following:

- Changing 'scoping' to 'systematic' throughout the document (Pg. 1-13).
- Including further details on the assessment of quality of included papers (Pg. 10).
- Updating the search prior to publication (Pg. 11).
- Changing the Inclusion/Exclusion criteria to PICO (Pg. 10).
- Providing further details on data synthesis (Pg. 12).

WAITING FOR ACCESS INTO TREATMENT "WAIT" STUDY: AN EXPLORATION OF CURRENT INTERVENTIONS OFFERED TO CHILDREN AND YOUNG PEOPLE ON CAMHS WAITING LISTS

PROTOCOL SYSTEMATIC REVIEW

**IRAS Project ID:**	284936
**Study Sponsor:**	University of Nottingham
**Sponsor reference:**	20059
**Funding Source:**	This study is funded by Emerging Minds (UKRI – ESRC), with support in kind from NIHR MindTech-MedTech Co-operative.
**Chief Investigator:**	Dr Charlotte Hall

Contents

SUMMARY OF PROTOCOL CHANGES 1
CONTENTS 3
STUDY PERSONNEL AND CONTACT DETAILS 4
OVERALL STUDY BACKGROUND AND RATIONALE FOR WAIT STUDY 5
WAITING TIMES: IMPACT 5
WAITING TIMES: DATA 5
IMPACT OF COVID-19 ON MENTAL HEALTH SERVICES 6
WAITING LIST INTERVENTIONS 7
RESEARCH QUESTION 1 7
RESEARCH QUESTION 2 7
RESEARCH QUESTION 3 8
THE EVIDENCE-BASE FOR WAITING-LIST INTERVENTIONS FOR CHILDREN AND YOUNG PEOPLE USING CHILD AND ADOLESCENT MENTAL HEALTH SERVICES: A SYSTEMATIC REVIEW PROTOCOL 8
AUTHORS ROLES AND RESPONSIBILITIES 8
ANTICIPATED START AND END DATES 8
STAGE OF REVIEW AT THE TIME OF PROTOCOL DEVELOPMENT 8
CONFLICTS OF INTEREST 8
INTRODUCTION 9
METHOD 9
1) Identify the question or objectives 9
2) Define inclusion and exclusion criteria 10
3) Methodological quality (Risk of bias) assessment 10
4) Search for evidence 11
4) Select evidence 11
5) Extract evidence 12
REFERENCES 13

STUDY PERSONNEL AND CONTACT DETAILS

Sponsor: University of Nottingham

Contact name: Ms Angela Shone
Research and Innovation
University of Nottingham
East Atrium
Jubilee Conference Centre
Triumph Road
Nottingham
NG8 1DH

Chief Investigator: Dr Charlotte Hall
Senior Research Fellow
Division of Psychiatry & Applied Psychology
Institute of Mental Health
University of Nottingham Innovation Park
Triumph Road
Nottingham
NG7 2TU
Email charlotte.hall@nottingham.ac.uk

Co-investigators:
Dr Althea Valentine (Study Co-ordinator)
Dr Bev Brown
Dr E Bethan Davies
Mrs Caitlin McKenzie
Miss Florence Day
Mr Nick Harrop
Ms Nikki Chapman
Dr Charlotte Sanderson
Prof Kapil Sayal
Prof Chris Hollis

OVERALL STUDY BACKGROUND AND RATIONALE FOR WAIT STUDY

Common mental health conditions in children and young people (CYP) include mood, anxiety, eating, disruptive, impulse-control, and conduct disorders. Neurodevelopmental disorders (e.g., intellectual disabilities, communication disorders, autism spectrum disorder [ASD], attention deficit hyperactivity disorder [ADHD], specific learning disorders, motor disorders, and tic disorders) are also a group of conditions with childhood onset often seen within CYP mental health services. These conditions have the potential to cause considerable impairment in functioning and wellbeing and affect every aspect of day-to-day life, including academic achievement and employment opportunities, quality of life, and peer and family relationships (Ogundele, 2018). Despite the impact of mental health conditions on CYP and their families, there is often a substantial delay in receiving assessments and treatment, especially as comorbidities often result in additional complexities in determining a diagnosis and subsequent treatment plan (Hansen, Oerbeck, Skirbekk, Petrovski, & Kristensen, 2018).

Waiting Times: Impact

Specialist Child and Adolescent Mental Health Services (CAMHS) teams are the outpatient service where children and young people (typically under 18-years old) in England, United Kingdom (UK), are referred to when they are experiencing severe, complex and enduring emotional, behavioural, or mental health difficulties. Referral pathways differ across Trusts but are usually via the CYPs general practitioner [GP] or school. Due to a difference in funding, eating disorders typically follow a different referral pathway than other mental health conditions, similarly, some Trusts also have different referral pathways for severe or life-threatening conditions such as psychosis, suicide, and self-harm, which necessitate immediate crisis assessment and for CYP with moderate to severe learning disability where care is provided for by specialist learning disability teams. CAMHS specialist services provide a multi-disciplinary team that can conduct assessments to look at whether the child meets the threshold for diagnosis, that is whether the child has a recognisable cluster of symptoms or difficulties over a specified time-period, which usually impact on daily functioning, and which are non-typical in terms of child development (specialist CAMHS services do not provide care for CYP who are experiencing a normal reaction to a significant life event). If a child has these symptoms or difficulties, which are usually observed during clinical interview and observation, the CAMHS team decide whether a clinical diagnosis would be helpful.

Once referred to CAMHS, CYP and their families often report long waiting lists for assessment, diagnosis, and treatment (Crenna-Jennings & Hutchinson, 2020; Hall et al., 2016)**.** Many CYP are not formally diagnosed (Michelson et al., 2011)**,** or clinicians may delay making a diagnostic decision (Hollis et al., 2018)**.** Diagnosis can help treatment planning and facilitate the integration of evidence-based interventions. However, there are various reasons why a formal diagnosis may not take place, including lack of clinical confidence (Hollis et al., 2018), training (Michelson et al., 2011)**,** or concerns on labelling and stigma (Martin, Fishman, Baxter, & Ford, 2011; Michelson et al., 2011). Additionally, once a diagnosis is made, a lack of adequately trained therapists (Cuenca et al., 2015) or availability of evidence based treatment, may further delay or even stop CYP receiving appropriate treatment.

Excessive waiting times are a barrier for accessing care and associated with negative outcomes. For example, Reardon, Harvey, Young, O'Brien, and Creswell (2018) interviewed parents of CYP with anxiety and found that waiting times had deterred them from seeking professional help. It is thought that waiting times may also contribute to clinical deterioration and increased risk for suicide or hospitalisation (Williams, Latta, & Conversano, 2008)**.** Waiting times may also be a barrier to engagement in treatment, with research indicated that the longer patients wait, the less likely they are to attend clinical appointments (Sherman, Barnum, Buhman-Wiggs, & Nyberg, 2009) and the more likely they are to drop out of therapy (Westin, Barksdale, & Stephan, 2014). But what exactly are the waiting times to receive treatment?

Waiting Times: Data

Reports on waiting time typically relied upon Freedom of Information (FOI) requests to CAMHS providers (NHS Trusts). For example, the Education Policy Institute (EPI) collated data for several years looking at the number of

children referred but not accepted for treatment, and on waiting times to assessment and treatment for children accepted by CAMHS. The EPI has shown that on average young people waited 56 days to start treatment. Although waiting times have decreased since 2015 (falling by 11 days from 2015 to 2019), the target of a 4-week wait (set in the government's 2017 green paper) was still not being met in 2019. Regional differences were identified with the longest median waiting times being in London (65 days) compared to the shortest being Midlands and East of England (49 days). The authors suggest that this is likely to be due to the higher number of accepted referrals in London, compared to other areas. Maximum waiting times indicated that some children were waiting longer than a year to access support (Crenna-Jennings & Hutchinson, 2020). This study can be commended for tracking waiting lists across England and looking at regional differences, however, the study did not look at differences in reason for referral on waiting times and availability of services.

Waiting time data can also be obtained through retrospective analysis of case notes. In an exploratory study looking at CAMHS waiting times in Scotland, UK during 2013/14, the authors conducted a retrospective analysis of referral data looking at referral source, reason, and outcome. Regression models were built to determine predictors of a referral being rejected and waiting time for referrals being accepted. Reason for referral was found to be a significant independent predictor of waiting time (Smith et al., 2018). Findings suggest that self-harm and eating disorders had the shortest waiting times and those referred for behaviours of hyperactivity and inattention waited significantly longer in CAMHS specialist services in Scotland. Whilst the researchers cannot conclude a reason for these discrepancies, they suggest that poorer future outcomes may result from delays in treatment.

More recently, the Mental Health Services Dataset (MHSDS) has been developed. Data is inputted locally but combined and published nationally by NHS Digital. The year of 2020/2021 is the third year that NHS Digital has produced data relating to waiting times in CYPMH. Data has improved during this time, for example, it now includes a specific code to record XenZone, an online provider of mental health services for CYP. It is also now more closely aligned with the key Children's Access to Mental Health Measure (MHS69). As such, changes over time from the dataset must be interpreted with caution. This is now the main dataset of the NHS. However, despite data being available, it lacks some variables that would be useful to consider, such as reason for referral, and it is also not inputted into by all Trusts. Arguably, the FOI approach may yield more fruitful data that is not routinely available and which can provide a representative, useful and reliable data set (Clifton-Sprigg, James, & Vujic, 2020).

Impact of COVID-19 on Mental Health Services

In December 2019, the first case of a novel virus was reported in Wuhan, China (World Health Organisation, 2020). With increasing levels of spread and severity, the World Health Organisation declared a pandemic in March 2020 and the UK entered a national lockdown, with schools, workplaces and all non-essential businesses closing (The Institute for Government, 2020). The first national lockdown ran between March and June 2020, a second between November and December 2020 and a third from January to March 2021. Between national lockdowns, contact restrictions remained. In the wake of the COVID-19 pandemic, it is likely that CYP mental health may have been impacted, with young people already experiencing mental health problems at greatest risk of decline (Fegert et al., 2020).

Most recent studies looking at CYP mental health, do not provide comparable pre-pandemic data, but one study, the Mental Health of Children and Young People in England report (2021) provides follow-up data from CYP (aged 6-23 years) surveyed in 2017 and prevalence estimates during the pandemic. Key findings showed that rates of probable mental disorder increased over time from one in nine (11.6%) to one in six (17.4%) of 6-16-year olds and one in ten (10.1%) to one in six (17.4%) of 17-19-year olds. Children and young people with probable mental health disorders were more likely than CYP without mental health disorders to have missed school and to have sleep problems, showing the impact of mental health difficulties on day-to-day life. Looking at changes in mental health over time in 6-16-year olds, more CYP experienced deterioration (39.2%) than improvement (21.8%) in mental health. This was greater in 17-23-year olds with 52.2% reporting deterioration and only 15.2% improvement in mental health. In terms of prevalence and access to CAMHS services, approximately 36.9% of children and young people with probable mental disorder accessed services in 2017 and only 29.5% accessed services in 2020. Many (45%) CYP with probable mental health conditions reported not seeking help due to the pandemic and lockdowns, a concern independently raised by clinicians. The study reports a sharp decrease in referrals to CAMHS during the lockdown. However, data are not directly comparable from 2017 to 2020 due to changes in the survey approach and it is not possible to delineate the effects of the pandemic and stress of lockdown, from other reasons for an increase in

mental health difficulties, therefore caution should be taken with comparing data directly. It does show that at the present time, most CYP wait longer than the 4-week period to start treatment, with only 20% of CYP starting timely treatment within a month of referral (Newlove-Delgado et al., 2021).

To date, no research has focused on understanding waiting times using routine clinic collected metrics, but existing research suggests that COVID-19 may have caused an increase in both mental health difficulties and difficulties in accessing services. The impact of the pandemic on service waiting times and referrals has not currently been investigated. Understanding how and where services are overstretched, will allow us to understand which mitigation strategies need to be implemented in order to support NHS services, and service users. For example, by providing interventions for those on waiting lists.

Waiting List Interventions

Whereas the majority of published research on "waiting lists" focusses on how to avoid missed appointments or how to manage diaries/booking in systems and triage systems more effectively, there is less evidence on how to actively support CYP on waiting lists. In recognition of this the James Lind Alliance (JLA) acknowledge developing CAMHS waiting-list interventions as a top-10 priority (http://www.jla.nihr.ac.uk/priority-setting-partnerships/Mental-health-in-children-and-young-people/downloads/Mental-Health-in-Children-and-Young-People-PSP-Supplementary-Report.pdf**)**. Despite this, there is currently no systematic review in this area and very few published papers looking at waiting list interventions for mental health. Indeed, a brief scoping review (conducted by our team) found no papers that directly looked at waiting list intervention for CAMHS.

There are, however, evidence-based interventions that may particularly lend themselves to being offered as waiting-list interventions. Such interventions may be delivered remotely, self-directed, or rely on minimal or asynchronous therapeutic contact. Remotely-delivered online interventions are increasing at a fast pace for example, in a systematic review looking at published papers from the period 2010-2016, Sutherland and colleagues (2018) identified 14 studies with a total of 284 participants with ASD across the lifespan. Telehealth services included assessments, early interventions, functional behavioural analysis, anxiety interventions, and language therapy. Although the studies varied in quality, the telehealth delivered services were comparable to face-to-face delivered services and better than control/comparison groups in experimental studies. More generally, there are a range of online interventions for CYP mental health issues which have shown some clinical benefit (see Hollis et al for review (Hollis et al., 2017)). Our discussions with CAMHS clinicians also reveal that some CAMHS may be being offered online interventions as waiting list treatments; however, there is no published evidence to support this at present.

There is therefore a need to understand the current waiting list interventions offered by CAMHS and explore issues around the barriers and facilitators to their use, as well as any evidence of benefits/impact on services resulting from their implementation.

RESEARCH QUESTION 1

The above research has led to the development of the first research question in this study, which has evolved over the course of the pandemic to additionally explore how the pandemic has affected waiting times in CAMHS. This study uses a FOI approach to ascertain waiting times over the past four years, including the pandemic year, to note any changes. In addition, the reason for referral and the route of referral is considered for the past two years to look at changes in reasons for referral during the pandemic. The first research question is:

1) What are the waiting times for CYP accessing treatment in Specialist CAMHS by reason for referral and region and has referral and waiting times been impacted on by the COVID-19 pandemic?

RESEARCH QUESTION 2

Once waiting times have been ascertained the study will move on to the second research question to explore what is being done within CAMHS services to help support CYP who are on waiting lists.

To date the focus of much research has been on reducing waiting times and there is no clear understanding of how often waiting-list interventions are being offered, their characteristics (i.e., leaflet/online) or evidence base.

The aim of the second research question is to understand the current provision of waiting list interventions for CYP accessing CAMHS. It is vital to understand this in order to facilitate and improve early intervention strategies. In addressing this we will identify which interventions may benefit from wider scale adoption across regions. We will also identify interventions that require future research to establish clinical and cost-effectiveness and gaps in early provision for specific disorders. The second research question is therefore:

2) What waiting-list interventions are currently offered to CYP by disorder and by region?
    - What are the barriers and facilitators to implementing waiting list interventions?
    - Has the provision of waiting list interventions been influenced by COVID-19?

RESEARCH QUESTION 3
Finally, the study explored the evidence-base for waiting-list interventions that are being implemented in routine care. Arguably, this would enable CYP to access timely support and may prevent further deterioration experienced on waiting lists. Accessing waiting list interventions may also result in service-efficiencies, such as: earlier symptom improvement; improved quality of life; requiring fewer sessions of the designated treatment; reducing the burden on healthcare systems and cost savings. The third research question:

3) What is the evidence-base for the available waiting-list interventions?

This protocol is developed to answer the final research question:

The evidence-base for waiting-list interventions for children and young people using child and adolescent mental health services: A systematic review protocol

Authors roles and responsibilities

- AZ Valentine (1st reviewer)
- S Hall (2nd reviewer)
- CL Hall (senior author and any discrepancies)
- Kapil Sayal (senior author)

Anticipated start and end dates

September 2021 to April 2022.

Stage of review at the time of protocol development

Preliminary searches completed.

Conflicts of interest

The authors declare that they have no known conflicts of interest.

Introduction

Part one of the WAIT study found that although waiting times are improving, they were still an issue and some children and young people (CYP) remained on waiting lists for a substantial time. The second part of this study explored what is being offered within CAMHS services to help support CYP who are on waiting lists. It was found that 'NHS approved apps', Kooth, Silvercloud, Blue Ice, Thrive and Togetherall were used as WLIs, as well as Goal Based Interventions (GBI), psychoeducation, guided self-help, risk management and support phone calls, online support, and increased staffing. Part three of the study was to conduct a systematic review to find out more about the body of evidence surrounding waiting list interventions.

The definition of WLI used in the survey (and throughout this review) was obtained using personal and public involvement (PPI) feedback about what young people and their families would consider a WLI. It stated:

> *"We define WLI as an intervention that is offered to children, young people and/or their families following acceptance of referral, but before the first appointment to CAMHS. WLI could be workshops, psychoeducation, online-delivered interventions, sign-posting to charities which deliver a target intervention, social prescribing (e.g. activities such as arts, music, sports, volunteering, gardening). The provision of leaflets with brief advice, signposting to apps, web-sites or charities that do not provide an intervention would not be considered WLIs".*

The reason for undertaking the systematic review is because we are interested in identifying what evidence is available and presenting an overview of the characteristics, nature, and diversity of the interventions within child mental health services. In particular, we are interested in noting the gaps in the research knowledge base and clarifying what the key concepts are in terms of WLIs, considering the outcomes clinically as well as in terms of user feedback on feasibility and acceptability and facilitators and barriers to engagement.

To date, as far as the researchers are aware, there have been no other existing systematic reviews, systematic reviews, or research syntheses of WLIs for children and young people accessing psychological services. A preliminary search for existing reviews was conducted on JBI Evidence Synthesis, Medline, PsychInfo, and Web of Science in September 2021, which revealed no relevant published papers (search "waiting list" AND "review" in title only). Although reviews have been conducted in general outpatient services (e.g. Caffery, Farjian, Smith, 2016 review of telehealth interventions for reducing waiting lists) no similar reviews in psychological services were found. In the Cochrane Database of Systematic Reviews, using the same search terms, several relevant trials were found but only one review which was also not relevant. Therefore, a systematic review was deemed the most appropriate review to be conducted at this stage.

The PRISMA and Centre for Reviews and Dissemination guidance for undertaking reviews in health care (Available from www.york.ac.uk/media/crd/Systematic_Reviews.pdf) were used as a framework for the protocol.

Method

1) Identify the question or objectives

Objective and research questions

The objectives of the systematic review were to:

(a) explore and summarise the available peer-reviewed evidence-base for interventions for CYP with mental health difficulties on waiting lists for mental health services;
(b) provide an overview of the characteristics, nature and diversity of the interventions; and
(c) explore the evidence in terms of outcomes of interest including clinical outcomes, service efficiencies and user impact, acceptability, and reported barriers and facilitators to engagement.

2) Define inclusion and exclusion criteria

The Population, Intervention, Comparators, and Outcomes (PICO) were used as a framework to guide the inclusion criteria and inclusion criteria are presented in Table 1.

Table 1: Inclusion and exclusion criteria

Population	Children and young people (up to 18 years) referred to a specialist mental health service for assessment or treatment of any mental health disorder. Participants may also be the families/carers or healthcare providers of these participants.
Intervention	We defined 'waiting list interventions', as an intervention used to support participants and/or their family whilst on a waiting list for mental health assessment, diagnosis and/or treatment. There were no restrictions on the frequency, timing, geographical location or healthcare setting, or those administering the intervention, but these details were noted in the data extraction as important features.<br><br>The following were excluded: waiting list management from a service perspective, models for healthcare delivery, appointment scheduling or improving access, opinions on general service satisfaction or clinical changes not linked to waiting list interventions, interventions that could be a waiting list interventions but are not tested as such, preventative trials not at the point of referral.
Comparators	The intervention must have been used to support participants whilst on a waiting list for assessment or treatment in a mental health service any comparators were considered.
Outcomes	Outcomes included clinical outcomes, service efficiencies and user impact, acceptability, and reported barriers and facilitators to engagement.
Types of sources of evidence	Evidence sources included qualitative and quantitative research studies and conference abstracts that reported clinical, cost-effectiveness, and/or perceptions (feasibility/usability). Non-peer review articles were excluded.

3) Methodological quality (Risk of bias) assessment

A rapid appraisal of the level of evidence will be assessed using the Oxford Centre for Evidence-Based Medicine (OCEBM; https://www.cebm.ox.ac.uk/resources/levels-of-evidence/ocebm-levels-of-evidence). The levels of evidence will be displayed in a table format with a range from 1-5, where 1 is the highest quality. One reviewer will conduct the quality appraisal, which will be verified by a second reviewer, any disagreements will be resolved via discussion, with the inclusion of a third person if necessary.

4) Search for evidence

A three-step search strategy will be used.

1) Search MEDLINE (Ovid) and PsycInfo, look at the first 10 relevant 'hits' text words (relevant terms from title/abstract/index).
2) Work with full research time to develop comprehensive list of search terms.
3) Conduct main search in MEDLINE (Ovid), PsycInfo, and Web of Science, with no restrictions on number of hits.

Data restricted by date from 2000 to 2022 to ensure interventions are relevant and by those published in the English language. The search will be repeated prior to publication in October 2023.

Design of study

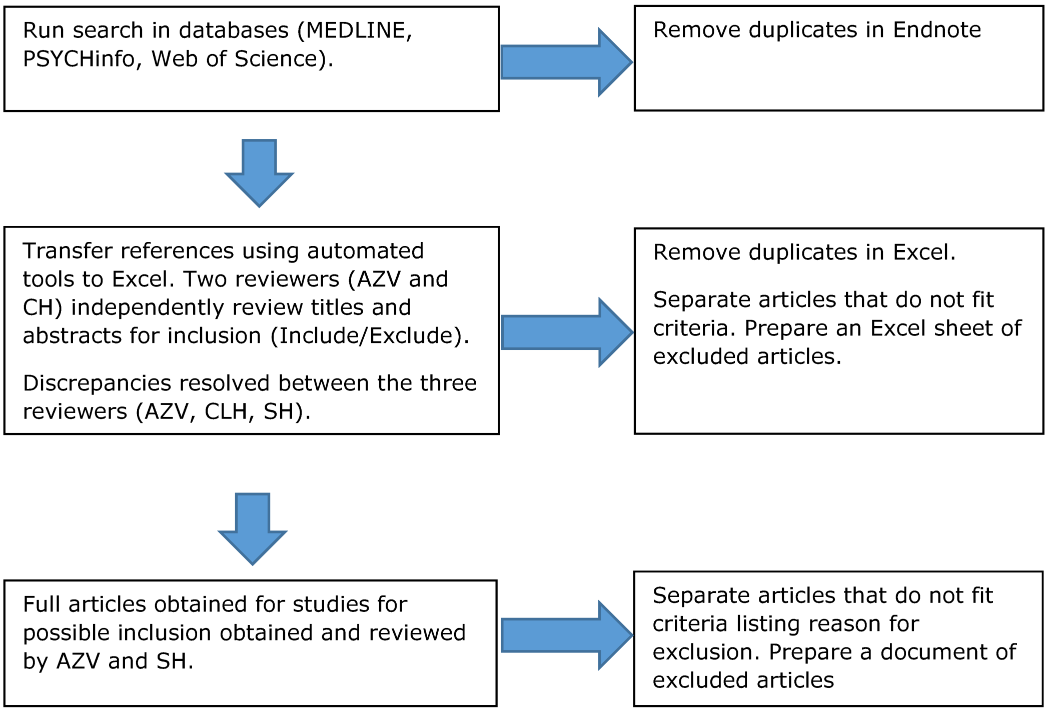

4) Select evidence

Data will be extracted into Endnote, which will be used to manage the results and remove duplicates. Pilot testing of the evidence selection will be conducted, with the first 25 titles and abstracts screened for relevance independently by the research team using the eligibility criteria. Minor amendments will be made to clarify terms, if necessary. When greater than 75% agreement is achieved within the research team, two researchers will examine the title and abstracts of the remaining papers for relevance independently. Retrieval of full texts will be conducted by one researcher and the details of the full text articles retrieved will be noted. Two researchers will review the full texts independently and the reason for exclusion will be noted (excluded papers Excel document). Disagreements will be resolved through discussion and mutual consensus. Following data extraction, backwards and forward citation chasing will be conducted using citationchaser to screen for additional relevant papers.

5) Extract evidence

A data extraction table was created (see below).

Stage 2: Data extraction

Study characteristics			
Name of 1st author and year	*Description of participants (age range, gender, conditions, population, country)*	*Aim and design/methodology*	*Type of intervention e.g. psychoed. and CBT and description of intervention*
Evaluation of study			
Clinical outcomes	*Service efficiency*	*User impact*	*Barriers and facilitators*

Data extraction: To avoid the risk of bias, standardized data extraction tools will be used to extract the data from the included reviews. The data extraction tool (Excel database) will be piloted before use prior to the main extraction by extracting the first 3 relevant papers and two reviewers clarifying any inconsistencies in the interpretation of fields. Any additions or modifications to the data extraction tool will be discussed and agreed upon by all researchers. One reviewer will extract the data and a second reviewer will verify this independently.

The data extraction tool will include: citation details, participant characteristics, description of the participants and main intervention(s), evaluation of the study including clinical outcomes, service efficiency, user impact and barriers and facilitators to engagement. The primary studies will not be obtained, and authors will not be contacted for further information.

6) Data synthesis

Data extracted from included studies will be analysed and summarised to address the review objectives using a formal narrative synthesis. The data will be synthesised firstly by a summary of included studies presented in tabular format. This will include information such as the study design, a description of the population (e.g., condition, age range, gender), details of the intervention (e.g., type, delivery, duration, to whom) and outcomes (clinical, service efficiencies, user impact). We will group the studies for synthesis in terms of intervention and condition. We will consider the type of intervention and duration, the aims of the papers, the methodology adopted, and the key findings relating to the outcomes of interest. The table will be supplemented with a discussion of the methodological and/or clinical differences across the studies, this will allow a greater understanding of the different types of interventions across different conditions.

Heterogeneity will be assessed informally by ordering the tables. It is anticipated that heterogeneity is likely to be substantial in terms of intervention types and subpopulations (e.g., age range, gender, condition). Due to the heterogeneity of the studies, the review will synthesise the data narratively rather than conduct a quantitative analysis.

7) Present results

In addition to producing a report for the funders of this review, a paper will be submitted to a leading journal in this field.

REFERENCES

Clifton-Sprigg, J., James, J., & Vujic, S. (2020). Freedom of Information (FOI) as a data collection tool for social scientists. *PLOS ONE, 15*(2), e0228392. doi:10.1371/journal.pone.0228392

Crenna-Jennings, W., & Hutchinson, J. (2020). Access to child and adolescent mental health services in 2019.

Cuenca, J., Glazebrook, C., Kendall, T., Hedderly, T., Heyman, I., Jackson, G., . . . Stern, J. (2015). Perceptions of treatment for tics among young people with Tourette syndrome and their parents: a mixed methods study. *BMC Psychiatry, 15*(1), 46. doi:DOI: 10.1186/s12888-015-0430-0

Fegert JM, Vitiello B, Plener PL, Clemens V. (2020). Challenges and burden of the Coronavirus 2019 (COVID-19) pandemic for child and adolescent mental health: a narrative review to highlight clinical and research needs in the acute phase and the long return to normality. Child Adolesc Psychiatry Ment Health, 12;14:20. doi: 10.1186/s13034-020-00329-3. PMID: 32419840; PMCID: PMC7216870.

Hall, C. L., Taylor, J. A., Newell, K., Baldwin, L., Sayal, K., & Hollis, C. (2016). The challenges of implementing ADHD clinical guidelines and research best evidence in routine clinical care settings: Delphi survey and mixed-methods study. *BJPsych open, 2*(1), 25-31.

Hansen, B. H., Oerbeck, B., Skirbekk, B., Petrovski, B. É., & Kristensen, H. (2018). Neurodevelopmental disorders: prevalence and comorbidity in children referred to mental health services. *Nordic journal of psychiatry, 72*(4), 285-291.

Hollis, C., Falconer, C. J., Martin, J. L., Whittington, C., Stockton, S., Glazebrook, C., & Davies, E. B. (2017). Annual Research Review: Digital health interventions for children and young people with mental health problems–a systematic and meta-review. *J Child Psychol Psyc, 58*(4), 474-503. doi:DOI: 10.1111/jcpp.12663

Hollis, C., Hall, C. L., Guo, B., James, M., Boadu, J., Groom, M. J., . . . Valentine, A. Z. (2018). The impact of a computerised test of attention and activity (QbTest) on diagnostic decision-making in children and young people with suspected attention deficit hyperactivity disorder: single-blind randomised controlled trial. *Journal of Child Psychology and Psychiatry, 59*(12), 1298-1308.

Martin, A.-M., Fishman, R., Baxter, L., & Ford, T. (2011). Practitioners' attitudes towards the use of standardized diagnostic assessment in routine practice: a qualitative study in two child and adolescent mental health services. *Clinical Child Psychology and Psychiatry, 16*(3), 407-420.

Michelson, D., Rock, S., Holliday, S., Myers, G., Tilki, S., Murphy, E., & Day, C. (2011). Improving psychiatric diagnosis in multidisciplinary child and adolescent mental health services. *The Psychiatrist, 35*(12), 454-459.

Ogundele, M. O. (2018). Behavioural and emotional disorders in childhood: A brief overview for paediatricians. *World journal of clinical pediatrics, 7*(1), 9.

Newlove-Delgado T, Williams T, Robertson K, McManus S, Sadler K, Vizard T, Cartwright C, Mathews F, Norman S, Marcheselli F, Ford T. (2021) Mental Health of Children and Young People in England, 2021. NHS Digital, Leeds.

Reardon, T., Harvey, K., Young, B., O'Brien, D., & Creswell, C. (2018). Barriers and facilitators to parents seeking and accessing professional support for anxiety disorders in children: qualitative interview study. *European child & adolescent psychiatry, 27*(8), 1023-1031.

Sherman, M. L., Barnum, D. D., Buhman-Wiggs, A., & Nyberg, E. (2009). Clinical intake of child and adolescent consumers in a rural community mental health center: Does wait-time predict attendance? *Community Mental Health Journal, 45*(1), 78-84.

Smith J, Kyle RG, Daniel B, Hubbard G. (2018). Patterns of referral and waiting times for specialist Child and Adolescent Mental Health Services. Child Adolesc Ment Health. Feb;23(1):41-49. doi: 10.1111/camh.12207. Epub 2017 Feb 9. PMID: 32677372.

Sutherland, R., Trembath, D., & Roberts, J. (2018). Telehealth and autism: A systematic search and review of the literature. *International Journal of Speech-Language Pathology, 20*(3), 324-336. doi:10.1080/17549507.2018.1465123

Westin, A. M., Barksdale, C. L., & Stephan, S. H. (2014). The effect of waiting time on youth engagement to evidence based treatments. *Community mental health journal, 50*(2), 221-228.

Williams, M. E., Latta, J., & Conversano, P. (2008). Eliminating the wait for mental health services. *The journal of behavioral health services & research, 35*(1), 107-114.
