# Supplementary material for: Waiting-list interventions for children and young people using child and adolescent mental health services: a systematic review
Source: BMJ Ment Health. 2024 Feb 1;27(1):e300844. doi: 10.1136/bmjment-2023-300844 (PMC10836350; doi:10.1136/bmjment-2023-300844)
Supplement: Supplementary data [file bmjment-2023-300844supp003.pdf]

Table 2: Summary of included studies

	STUDY										POPULATION																		INTERVENTION																				OUTCOMES			
**1st Author (year) and country**	**DESIGN**	Feasibility	RCT	Quasi Experimental	Action Research	Qualitative	Service evaluation	Mixed methods	**QUALITY**	Level of evidence	CONDITION	Self-harm/suicide	ASD	Behavioural	Anxiety/depression	Eating disorder	Transgender health	Generic	**AGE**	Under 5 years	5-11 years	12-16 years	17-18 years	GENDER	Mixed	Mixed – male	Mixed – female	No. of CYP participants	**TYPE**	Coaching	Psychoeducation	Parent support	Self-help	Other organisations	**DELIVERY**	Face-to-face	Videoconference	Telephone	Video/online/app	**DURATION**	Single session	2-5 sessions	6-12 sessions	> 12 sessions	**TO WHOM**	Parent only	CYP only	Both parent and CYP	**EVALUTAION**	Clinical	Service efficiency	User impact
**Beard (2022) USA**		X								4					X						X	X			NR	NR	NR	14			X	X	X						X							X				X		X
**Bernie (2022) Australia**		X	X							3			X							X	X					X		16		X						X	X	X				X				X				X		X
**Bruett (2022) USA**							X			5						X				NR	NR	NR	NR		NR	NR	NR	268			X	X	X	X			X					X				X					X	X
**Ching (2022) UK**		X						X		3								X			X	X	X		X			29			X		X						X		X						X	X		X		X
**Connolly (2013) Ireland**					X					4			X							X					NR	NR	NR	4			X	X				X						X				X						X
**Couturier (2023) Canada**		X		X						3						X					X	X	X				X	30			X		X						X			X				X				X		X
**Dababnah (2023) USA**				X						3			X	X						X	X							15			X	X					X						X			X				X		
**Dahlgren (2021) Australia**								X		4							X				NR	X	X		X			276			X		X	X		X					X						X			X	X	X
**Eade (2018) Australia**							X			4							X				X	X	X		NR	NR	NR	194			X	X	X	X		X					X							X			X	
**Johnston (2004) UK**							X			4				X							X					X		34			X	X				X					X	X	X			X						
**Kunze (2021) USA**			X							4			X							X					X			6		X	X						X						X					X		X	X	X
**Loucas (2020) UK**		X	X							3					X							X	X				X	24			X		X			X		X				X					X			X		X
**McGarry (2008) Ireland**			X							2								X		X	X	X				X		60			X	X	X			X					X	X				X				X		X
**Rivard (2017) Canada**						X				4			X							X						X		94		X	X					X								X		X				X		X
**Spettigue (2015) Canada**				X						4						X											X	36			X	X	X			X		X	X					X		X				X		X
**Terry (2003) UK**		X								5								X		X	X	X			X			12			X	X				X					X	X						X		X		X
**Wade (2022) Australia**		X								3						X						X						16			X	X	X			X	X		X				X			X				X	X	X
**White (2023) USA**				X						3		X								NR	NR	NR	NR				X	125			X	X				X						X	X	X				X		X		X
Total	-	7	4	4	1	1	3	2	-	-	-	1	5	2	2	4	2	3	-	7	9	9	5		4	4	4	1253		3	17	11	10	3		12	5	3	5		6	9	5	3		11	3	5		15	5	16

Notes: RCT=randomised controlled trial, ASD=autism spectrum disorder, Mixed fe/male=predominantly fe/male, CYP=children and young people, NR=not reported.
