# Supplementary material for: Waiting-list interventions for children and young people using child and adolescent mental health services: a systematic review
Source: BMJ Ment Health. 2024 Feb 1;27(1):e300844. doi: 10.1136/bmjment-2023-300844 (PMC10836350; doi:10.1136/bmjment-2023-300844)
Supplement: Supplementary data [file bmjment-2023-300844supp002.pdf]

Supplementary Table A1: Summary of included studies

Surname of 1st author, year and brief description of study	Evaluation of study	Conditions[a], N, Gender[b], Population[c], Age, Country
**Beard et al. (2022)**<br>**Aim:** Test a mobile app-based intervention using health interpretation bias in parents of anxious young people.<br>**Design/methodology:** Cohort study<br>**Intervention:** HabitWorks, 4-week smartphone delivered interpretation bias intervention for parents (with additional access for subsequent 4-week FU period).	**Clinical outcomes:** There was a significant decrease in parent anxiety as measured on GAD-7 from baseline (mean=6.93, SD=3.73) to post-treatment follow-up (mean=4.43, SD=2.62) and 1- month follow-up (M = 3.64, SD = 2.76). Repeated measures ANOVA (with Greenhouse-Geisser correction) showed significant decrease in anxiety symptom severity (F(1.28, 16.638) = 5.816, p = .021, g2 = 0.309), post-hoc tests suggest this was significant from baseline to end of treatment. No related adverse events.<br>**Service efficiency:** NR<br>**User impact:** Rated as being moderately creditable. HabitWorks met a priori benchmarks for adherence and acceptability and satisfaction. Usability was rated as excellent.<br>**Barriers and facilitators:** Some participants said that the lack of customisation and repetitiveness or irrelevancy of topics was a barrier to completion.	**Condition:**<br>Parents of CYP on waiting list anxiety<br>**Population:**<br>Parents<br>N= 14 parents<br>(87.5% of eligible participants)<br>**CYP Gender:**<br>NR CYP Parents 2m, 12f<br>**CYP Age:**<br>8-16 years (parent 36-50)<br>**Country:**<br>USA
**Bernie (2022)**<br>**Aim:** Determine whether short-phase Occupational Performance Coaching (OPC) alongside service navigation support is feasible and beneficial for families waiting for an autism assessment.<br>**Design/methodology:** A pilot feasibility study<br>**Intervention:** Intervention delivered face-to-face or videoconferencing (online) and involved 4 sessions (lasting between 35-70mins) of a manualised OPC intervention.	**Clinical outcomes:** 75% of participants increased scores in functional goal attainment effect size=13.302; measured by the Canadian Occupational Performance Measure, COPM) compared to 25% of treatment as usual (TAU) control. Confidence intervals were not reported. Initial positive trends for decreased parental stress index (PSI) scores in the video-conference arm (change score=-14.5).<br>**Service efficiency:** Refers to the study being cost-neutral for participants.<br>**User impact:** Primary outcomes were in terms of recruitment (9% of eligible families) and retention (75%, 4 families withdrew [3 usual care; 1 face-to-face]). High ratings of intervention acceptability and practicality from parent reports. All questionnaire responders (N = 6, 75%) reported that they were satisfied with the intervention. All participants were happy with videoconferencing, and 66% in face-to-face coaching were uncertain whether the location was a good fit. Most participants (n = 5, 83%) would recommend the intervention to others.<br>**Barriers and facilitators:** Two participants required translators; both completed the study but the feasibility for families with English as a second language was not assessed. Large variation in accessing services at baseline and through the study period.	**Condition:**<br>Autism Spectrum Disorders<br>**Population:**<br>Parents<br>N=16 families<br>(9% of eligible participants)<br>**CYP Gender:**<br>Mixed[m]<br>11m, 5f<br>**CYP Age:**<br>0-7yr<br>**Country:**<br>Australia
**Bruett et al. (2022)**<br>**Aim:** Support families on waiting list for eating disorders.<br>**Design/methodology:** Service evaluation of implementation (quality improvement not research).<br>**Intervention:** Family-Based Treatment (FBT), implemented by a tertiary specialist adolescent ED service. Tailored to family's need, including	**Clinical outcomes:** NR.<br>**Service efficiency:** *Individual sessions:* reduced burden on staff by providing treatment for families who may not need to go on to receive long-term treatment, potentially reducing waiting lists; no data provided. Increased efficiency of the appointments, however, no data given. *Workshops: not stated.*<br>**User impact:** *Individual sessions:* Families were grateful to have consultation whilst waiting. *Workshops:* Caregivers were very positive. Felt better able to support their child. Some felt overwhelmed by listening to others' experiences. *Caregiver support group:* enjoyed speaking to other parents in similar situation.<br>**Barriers and facilitators:** *Individual sessions:* Burden on administration staff to organise appointments, this was a significant barrier. The consultant needs considerable experience of eating disorders to undertake the	**Condition:**<br>Eating disorders<br>**Population:**<br>Parents and children/young people<br>*Individual sessions:* 268 referred and 55% completed a visit<br>*Workshops:* 66 families<br>*Support group:* 20 families<br>**CYP Gender:**

Surname of 1st author, year and brief description of study	Evaluation of study	Conditions[a], N, Gender[b], Population[c], Age, Country
psychoeducation, behavioural support. Delivery included individual sessions (1-4 visits, 30 mins), workshops (4 weeks, each week 90 mins) and caregiver support group (1 hour, fortnightly).	session. *Workshops:* low resources to set-up. Largest challenge was keeping caregivers engaged and attending all 4 workshops. *Caregiver support group:* difficulties scheduling families to get together.	NR<br>**CYP Age:**<br>Up to 25 years, lower age NR<br>**Country:**<br>USA
**Ching (2022)**<br>**Aim:** Assess the feasibility and impact of a remote appointment single-session intervention (SSI), which is a digital growth mindset WLI for CYP awaiting mental health service support.<br>**Design/methodology:** A mixed methods case series design pilot study.<br>**Intervention:** The intervention was a 20-30-minute self-administered online SSI. Patients were able to complete the intervention with or without their caregiver depending on their needs.	**Clinical outcomes:** Negligible improvements were seen across measures at the 1-month follow-up. A small to moderate effect size improvement was noted in personality mindset post-treatment [IPT-Q (d = 0.54)], which increased substantially at 3-month FU [IPT-Q (d = 1.26)]. No effect was found for perceived control [PCSC (d =_0.08)] or secondary control [SCSC (d = _0.04)] post-treatment with a small to moderate improvement at 3-month FU [PCSC (d = 0.19); SCSC (d = 0.53)]. Small to moderate effects were also seen for generalised anxiety [(d = 0.45)], panic [(d = 0.29)], obsessions/compulsions [(d = 0.18)] at 3-month follow-up.<br>**Service efficiency:** NR<br>**User impact:** Most participants found the intervention enjoyable and accessible and felt that it gave them hope, but some male adolescents did not find the intervention interesting. Improvements were suggested such as wanting greater diversity in the ages and difficulties presented in the stories to ensure they were relatable to a variety of issues CYP faced.<br>**Barriers and facilitators:** Patients liked having transcript/slides and audio narration, especially those with attention/learning disabilities, some CYP liked the stories of similar CYP which they found valuable and relatable, others found the stories to be inauthentic and/or unrelatable. Flexible delivery was possible, with caregiver support provided as necessary.	**Condition:**<br>Generic<br>(complex physical/mental health needs)<br>**Population:**<br>Children and young people<br>N=29 CYP<br>(74% of eligible participants) 25 completed intervention<br>**CYP Gender:**<br>Mixed<br>14m, 11f<br>**CYP Age:**<br>8-18yr<br>**Country:**<br>UK
**Connolly (2013)**<br>**Aim:** Evaluate a short-term parent education programme used as WLI in a multi-disciplinary therapeutic service for families of children waiting for an autism assessment.<br>**Design/methodology:** Final evaluation stage was relevant for this review, which used a qualitative approach including focus groups and questionnaires.<br>**Intervention:** Intervention delivered face-to-face and involved 4 sessions (lasting 2.5 hours) delivered once a week over a 4-week period, including refreshments and informal chat followed by psychoeducation. Sessions were designed based on the outcomes of an earlier focus group study about what parents wanted.	**Clinical outcomes:** NR<br>**Service efficiency:** NR<br>**User impact:** Parents described feelings of hope and calm, confidence and being respected in the group. Parents were reassured by their increased understanding of the diagnostic process and the nature of ASD and had increased confidence in their own abilities.<br>**Barriers and facilitators:** Most parents would not change the programme. Suggestions for improvements to the programme included more visual and video input about the implementation of strategies, having more parents in the group and having clear end times.	**Condition:**<br>Autism Spectrum Disorder<br>**Population:**<br>Parents<br>N=5 parents (4 families)<br>(20% of eligible participants)<br>**CYP Gender:**<br>NR<br>**CYP Age:**<br>2-5yr<br>**Country:**<br>Ireland

Surname of 1st author, year and brief description of study	Evaluation of study	Conditions[a], N, Gender[b], Population[c], Age, Country
**Couturier (2022)**<br>**Aim**: Evaluate an adapted guided self-help family-based treatment (GSH-FBT) model with no therapist involvement.<br>**Design/methodology:** Feasibility study using an interrupted time series (ITS) design (quasi-experimental approach)<br>**Intervention:** The intervention was parental self-help available over a 2-week period (with additional access for subsequent 6-week FU period) including 11 pre-recorded online videos and reading material with the aim to empower parents to renourish their child and interrupt ED behaviours.	**Clinical outcomes:** Significant increase in weight gain after intervention, especially in the subgroups with lower weight at baseline (average weight gain 0.24 lbs/week pre-intervention, increased significantly to 0.78 lbs/week post-intervention (p < .034)) and increase in parental self-efficacy (17.6 to 20.7 on the PvED scale, t 4.35, p < .001, df 28) with a large effect size (Cohen's d 0.81, 95% CI 0.38–1.22).<br>**Service efficiency:** NR<br>**User impact:** Most participants (93.3%) completed the online course and watched all videos. Authors report that the intervention allowed greater flexibility and convenience due to being delivered online<br>**Barriers and facilitators:** From a waiting list of 97 families, 32 parents expressed an interest. The authors suggest parents who believed in the efficacy of self-help were more likely to participate and therefore more likely to benefit.	**Condition:**<br>Eating Disorders<br>**Population:**<br>Parents<br>N=30 parents<br>(31% of eligible participants)<br>**CYP Gender:**<br>Mixed[f]<br>26f, 3m<br>**CYP Age:**<br>7-17yrs<br>**Country:**<br>Canada
**Dababnah et al. (2023)**<br>**Aim: E**xamine short-term impact of peer-to-peer psychoeducational intervention for Black children and families awaiting diagnostic assessment.<br>**Design/methodology:** Cohort study<br>**Intervention:** Adapted version of Parents Taking Action (PTA) psychoeducational programme delivered by trained peer, one-to-one online over 12 sessions, including topics on supporting communication and understanding child development.	**Clinical outcomes:** The family outcome total score (F [2, 27] = 8.60, p = 0.00), significantly improved pre-post intervention, with medium to large effect sizes (d = 1.06). Three sub-scales “understanding child’s strengths, needs, and abilities”; “knowing rights and advocating for child”; and “helping child develop and learn” outcomes significantly improved between preintervention and postintervention, with medium to large effect sizes (d = 0.51–0.83). Changes in child behaviour and parenting stress were not statistically significant., and the effect sizes were small to very small. Parent depression significantly decreased at pre-post intervention, with a medium effect size (d = -0.51).<br>**Service efficiency:** NR.<br>**User impact:** NR.<br>**Barriers and facilitators:** NR.	**Condition:**<br>Autistic spectrum disorders/development<br>**Population:**<br>Parents<br>N=19 families received intervention<br>(15 completed outcome measures)<br>(27.1% of eligible participants)<br>**CYP Gender:**<br>11m, 4f<br>**CYP Age:**<br>2-8 years (mean 4.6 yrs)<br>**Country:**<br>USA
**Dahlgren (2021)**<br>**Aim:** Explore whether a First Assessment Single-Session Triage (FASST) clinic is able to positively impact patients’ mental health and well-being for CYP who are TGD.<br>**Design/methodology:** A convergent, parallel mixed methods study including 1) a one-group pre/post study using clinical surveys, 2) a non-	**Clinical outcomes:** Parent-rated Child Behavior Checklist (CBCL) T scores decreased for both depressive and anxiety problems (standardized mean difference [SMD] 5 0.24; 95% CI: 0.36 to 0.11; P < .001 and SMD 5 0.14, 95% CI: 0.26 to 0.02; P 5 .025, respectively). Patient quality of life also improved, Child Health Utility 9D (CHU9D) increase in mean scores from 0.41 to 0.51 (SMD 5 0.39, 95% CI: 0.23 to 0.56; P < .001). The proportion of families in the unhealthy family functioning range reduced by 11.0% (55.8% vs 44.9%; 95% CI: 21.4 to 0.6; P 5 .039). Comparison with historic controls showed reduced depression (SMD 5 _0.24; 95% CI: _0.50 to 0.01; P 5 .065) and anxiety (SMD 5 _0.31; 95% CI: _0.57 to _0.05; P 5 .021). The qualitative study described those who had taken part in FASST with an improved outlook, validation, sense of self and confidence.	**Condition:**<br>Transgender health<br>**Population:**<br>Children and young people<br>N=142 FASST<br>N=120 control<br>N=14 interviews<br>(NR % of eligible participants)<br>**CYP Gender:**

Surname of 1st author, year and brief description of study	Evaluation of study	Conditions[a], N, Gender[b], Population[c], Age, Country
equivalent study with a historical control group, 3) qualitative interviews.<br>**Intervention:** The intervention was a single-session face-to-face waitlist clinic (FASST clinic). A Home, Education, Activities, Drugs, Sexuality and Suicide (HEADSS) youth psychosocial assessment was undertaken, and information, education, and support provided. The clinic was led by a clinical nurse consultant.	**Service efficiency:** After FASST, there was minimal difference in health professional use apart from an increase in the number of visits to general practitioners (D 5 0.33; 95% CI: 0.18 to 0.48; P < .001). Antidepressant use was also similar post-FASST, but there was significantly greater use of medications for menstrual suppression among birth-assigned female individuals (D 5 35.0%; 95% CI: 24.0 to 46.9; P < .001).<br>**User impact:** Interviewed participants reported positive feedback about FASST.<br>**Barriers and facilitators:** NR.	*FASST study*<br>Mixed<br>105f, 37m<br>80 TGD male<br>29 TGD female<br>16 nonbinary<br>16 unsure<br>1 NR<br>**CYP Age:**<br>NR – under 18<br>*Control study*<br>Mixed<br>61f, 59m<br>**CYP Age:**<br>NR – under 18<br>**Interviews**<br>Mixed[f]<br>13f, 1m<br>12 TGD male<br>1 TGD female<br>1 unsure<br>**CYP Age:**<br>13-17yrs<br>**Country:**<br>Australia
**Eade (2018)**<br>**Aim:** Document the creation and implementation of a single-session nurse-led assessment clinic (SSNac) in gender services.<br>**Design/methodology:** Service evaluation of single-session nurse clinic using wait time as the outcome.<br>**Intervention:** The SSNac involved a 90-minute, face-to-face, single-session consultation by the clinical nurse consultant for TGD patients and their primary caregiver(s). This included a 30–40-minute biopsychosocial assessment with the	**Clinical outcomes:** NR<br>**Service efficiency:** The introduction of SSNac included an additional 6 appointments each week. Outcomes of the SSNac - wait time reduced from 14 to 4 months with a more efficient and effective triage system whereby patients requiring urgent triage are seen for multidisciplinary assessment for a medical pathway within 2 months. Of the 194 patients attending SSNac, 8.2% were discharged, 12.9% were fast-tracked and 78.9% of patients followed routine assessments.<br>**User impact:** NR<br>**Barriers and facilitators:** NR	**Condition:**<br>Transgender health<br>**Population:**<br>Parents and children/young people<br>N=194<br>(NR% of eligible participants)<br>**CYP Gender:**<br>NR<br>**CYP Age:**<br>8-17yrs<br>**Country:**<br>Australia

Surname of 1st author, year and brief description of study	Evaluation of study	Conditions[a], N, Gender[b], Population[c], Age, Country
CYP to identify an appropriate triage pathway and provide health information and immediate interventions. This was followed by a joint consultation with the primary caregiver(s), including further education and links to community-based services relevant groups. The clinic was led by a clinical nurse consultant.		
**Johnston (2004)**<br>**Aim:** Evaluate a pilot waiting list intervention involving a health visitor with additional training in behaviour management, providing support for families with children with behavioural difficulties on the waiting list for Tier 3 CAMHS<br>**Design/methodology:** Service evaluation (parent rating scale and free text responses).<br>**Intervention:** Intervention involved face-to-face support which drew on elements of social learning theory, behaviour modification, parent training and solution-focused therapy. On average families were seen for four sessions, including the assessment (range 1-11). The clinic was led by a Health Visitor (with regular clinical supervision from a consultant clinical psychologist from the Tier 3 CAMHS team).	**Clinical outcomes:** SDQ data was only available at assessment and follow-up for 15 children. There were significant reductions on conduct, emotional and hyperactivity sub-scales, the effect size for the reduction of the total score of the SDQ was 0.9 and the impact score reduction 1.1. which demonstrates a positive effect of WLI.<br>**Service efficiency:** Low drop-out rate compared to CAMHS (12% vs 25%). The service was deemed to be successful, and a full-time permanent post was created in the service.<br>**User impact:** 19 families completed the feedback questionnaire and these families were highly satisfied (4.8/5 average) with the intervention. Suggested improvements included reducing the waiting list, meeting others with the same problems and being able to contact an advice line whilst waiting for an appointment.<br>**Barriers and facilitators:** NR.	**Condition:**<br>Behavioural<br>**Population:**<br>Parents<br>N=34 families<br>(89% of eligible participants)<br>**CYP Gender:**<br>Mixed[m]<br>28m, 6f<br>**CYP Age:**<br>5-11yrs<br>**Country:**<br>UK
**Kunze (2021)**<br>**Aim:** Examine the effects of a parent-mediated intervention to in/decrease child in/flexibility and the parent's use of applied behaviour analytic (ABA) strategies during play.<br>**Design/methodology and aim:** Single-case experimental design study<br>**Intervention:** Telehealth ABA intervention with parent/child in their home and researcher in a private office. The intervention phase began with an education session including the four ABA intervention strategies modelling,	**Clinical outcomes:** Tau-U was calculated to statistically estimate the amount of change following the WLI, this showed a strong effect between the intervention and parent strategy use and a general increase in flexible behaviours and a decrease in inflexible behaviours. The last five sessions of the intervention were when all parents used the highest number of strategy sequences and children showed their highest levels of flexible and lowest levels of inflexible behaviours. Inflexible behaviours were individual to each participant and included negative responses (e.g., whining, screaming, hitting); repetitive movements (e.g., flipping, mouthing, throwing toys) or interests (e.g., playing with only one toy). Flexible behaviours included exchanging materials and toys, verbally or physically acknowledging the parent during play, and changing play focus to new ideas. Findings were consistent across participants with one exception demonstrating a moderate effect for flexible behaviours and a strong effect for inflexible behaviours.<br>**Service efficiency:** NR. Only the costs of the intervention were reported, the play materials cost $125.68 US dollars per child. Families kept the toys after the study.	**Condition:**<br>Autistic spectrum disorders<br>**Population:**<br>Parents and children/young people<br>N=6 parent/child dyads<br>**CYP Gender:**<br>Mixed<br>2m, 4f<br>**CYP Age:**<br>1.5-3y<br>**Country:**<br>USA

**Surname of 1st author, year and brief description of study**	**Evaluation of study**	**Conditions[a], N, Gender[b], Population[c], Age, Country**
prompting, differential reinforcement of alternative behaviours, and response interruption and redirection (RIRD) and a coaching session including immediate feedback and error correction. Sessions 2-15 involved twice weekly 40-minute sessions, including watching behaviour and coaching across a 12-week period. Sessions facilitated by a behaviour analyst with 25 years of experience.	**User impact:** Participants rated the intervention as highly effective and usable.<br>**Barriers and facilitators:** A parent with strong play arrangement skills could increase flexibility with the least intrusive sequence.	
**Loucas (2020)**<br>**Aim:** Assess feasibility, acceptability, and preliminary outcomes of the DISCOVER intervention for 15-18-year-olds with anxiety and/or depression<br>**Design/methodology:** Feasibility RCT.<br>**Intervention:** DISCOVERY intervention involves a one-day (6.5 hours) group workshop format with up to 4 individual phone calls (20-30mins) to follow-up. The workshop followed a manualised CBT content focusing on problem- and emotion-focused coping skills, Sessions were co-facilitated by two doctoral-level clinical psychologists.	**Clinical outcomes:** Trends noted on anxiety and mental well-being, but not depression however no conclusive differences were noted. Confidence intervals were wide and the sample size was small (17 intervention, 7 control).<br>**Service efficiency:** NR<br>**User impact:** Attendance in the one-day group workshop was high and high levels of satisfaction from those who agreed to take part, however, there were difficulties in recruitment.<br>**Barriers and facilitators:** The WLI could be improved by providing information to address CYP concerns about the group workshop format, offering choice about preferred contact for follow-up (face-to-face or phone) and potentially offering to CYP with anx/dep who do not meet CAMHS referrals criteria.	**Condition:**<br>Anxiety/Depression<br>**Population:**<br>Children and young people<br>N=24<br>(24.7% of eligible participants).<br>**CYP Gender:**<br>Mixed[f]<br>21f, 3m<br>**CYP Age:**<br>15-18yr<br>**Country:**<br>UK
**McGarry (2008)**<br>**Aim:** Evaluate the effectiveness of a brief consultation and advice **(**BCA) approach<br>**Design/methodology:** A two-group repeated measures randomised controlled design study<br>**Intervention:** The face-to-face intervention used a brief consultation and advisory (BCA) model, a collaborative, client-centred way of working with families, modified and evaluated for CAMHS. The session lasted 1-2 hours and families were given the option for a further session 2 weeks later, and a third session 2-3 months later.	**Clinical outcomes:** The BCA model was found to be significantly more effective than TAU at 6 months, as evidenced by a statistically significant continued improvement in SDQ over time.<br>**Service efficiency:** NR<br>**User impact:** Although there was no negative impact due to being on the waiting list for the TAU group, more parents dropped out in the TAU group than in the BCA group, suggesting that the longer a child has to wait for their first appointment, the more likely they are to drop out either before being seen or mid-treatment. Both groups were highly satisfied with the treatment received with no significant differences across groups. Most parents in the BCA group (94%) were happy they received this treatment and 41% in the TAU wanted to receive BCA. BCA parents reported feeling empowered to make changes and appreciated not waiting for support.<br>**Barriers and facilitators:** BCA model is only applicable for certain patients and requires adequate screening to assess for suitability.	**Condition:**<br>Generic<br>**Population:**<br>Parents<br>N=60<br>(23.2% of referrals)<br>**CYP Gender:**<br>Mixed[m]<br>44m, 16f<br>**CYP Age:**<br>3-16yrs<br>**Country:**<br>Ireland
**Rivard (2017)**	**Clinical outcomes:** Parenting stress levels increased over the 12-month period - large main effect of time on parenting stress, $F(1, 62) = 10.77$, $p = .002$, $\eta 2 = .15$. Parent's gender had a moderate main effect on	**Condition:**<br>Autistic spectrum disorders

Surname of 1st author, year and brief description of study	Evaluation of study	Conditions[a], N, Gender[b], Population[c], Age, Country
**Aim:** Evaluate parental satisfaction with the content and format of the social validity of a training and coaching program for parents on WL, as well as perceived effects on family, and parental stress **Design/methodology and aim:** Qualitative. **Intervention:** Five intensive face-to-face group training sessions led by a psychologist with EBI training, followed by individual, at-home coaching for one hour each week over the next 12 months, including strategies to cope with their child's disorder based on applied behavioural analysis (ABA) that may facilitate everyday life, parent-child interactions and routines and introducing the Picture Exchange Communication System (PECS).	parenting stress, $F(1, 62) = 6.64$, $p = .012$, $\eta 2 = .10$, with mothers reporting more stress than fathers, but fathers experienced a greater increase in stress levels over the duration of the program. **Service efficiency:** NR. **User impact:** The program had positive effects on parents' psychological well-being (42.6 %), their family's quality of life (42.6 %), their parenting skills (38.3 %) and their child's behaviour of which most parents (94.6 %) reported positive effects. 45% requested more frequent/intensive/longer interventions and 27% requested improved accessibility including an additional language other than French. **Barriers and facilitators:** NR	**Population:** Parents N=94 parents **CYP Gender:** Mixed[m] 78m, 16f **CYP Age:** 2-5yr **Country:** Canada
**Spettigue (2015).** **Aim**: Whether a psycho-education session combined with bi-weekly telephone calls, could improve parents' ED knowledge, and self-efficacy for helping their child recover, decrease ED impact on the family, and improve ED symptoms. **Design/methodology and aim:** Randomized controlled study **Intervention:** An initial 2-hour psycho-educational face-to-face session delivered by a psychiatrist and physician specialising in ED treatment, including psychoeducation. Subsequent bi-weekly telephone calls with an experienced senior nurse (up until the time of formal assessment, average 94-day wait) aimed to provide support to parents. Families offered free books on ED, a meal-support DVD and a list of other recommended resources (i.e., recommended books, articles, websites, and local support groups).	**Clinical outcomes:** Increase in parents' general knowledge about eating disorders - the significant main effect of knowledge across time, Wilks' Lambda = .54, $F(2,25) = 10.85$, $p < .001$, partial $\eta 2 = .465$,. Significant interaction effect between group and self-efficacy across time, Wilks' Lambda = .58, $F(2, 28) = 10.10$, $p < .001$, partial $\eta 2 = .419$. Pair-wise comparisons reveal that self-efficacy did not differ between groups at baseline, $F(1,29) = .866$, $p = .354$, partial $\eta 2 = .030$, but the intervention group reported significantly higher feelings of self-efficacy at Time 2, $F(1,29) = 24.14$, $p < .001$, partial $\eta 2 = .454$, and at assessment (Time 3), $F(1,29) = 6.27$, $p = .018$, partial $\eta 2 = .178$. No group differences in terms of eating disorder symptom impact. **Service efficiency:** NR. **User impact:** Parents were very satisfied with the quality of the workshop ($M = 3.91$, $SD = 0.29$), found the information very helpful ($M = 3.73$, $SD = 0.46$), and would recommend to other families ($M = 3.91$, $SD = 0.29$). Six parents were interviewed about user impact, they felt that the information they received made them realize how ill their child was and that this was a good thing. They were reassured that people could help and found the intervention useful, making them feel more empowered, less guilty, helpless, and hopeless. **Barriers and facilitators:** NR	**Condition:** Eating disorders **Population:** Parents N=51 parents (27.1% of eligible participants) **CYP Gender:** 36 CYP assessed Mixed[f] 35f, 1m **CYP Age:** Mean 15.6yr, SD 1.47 **Country:** Canada

Surname of 1st author, year and brief description of study	Evaluation of study	Conditions[a], N, Gender[b], Population[c], Age, Country
**Terry (2013)**<br>**Aim:** Reduce the waiting list and provide a brief intervention for those waiting<br>**Design/methodology:** A pilot feasibility study in CAMHS.<br>**Intervention:** Referrals were invited to 1-hour face-to-face appointments which were jointly facilitated by two clinicians (nurse specialist, counsellor, psychiatrist or social worker), who started brief intervention work with the family (up to a maximum of 4 follow-up sessions). The aim of the sessions was solution focused.	**Clinical outcomes:** The case study reported improved child behaviour and functioning, but no supporting data were provided.<br>**Service efficiency:** 10 families attended the WLI. Two families needed the initial appt only and no further work; a further two attended the initial appt and required one follow-up session and were then discharged, a further three continued to attend for short-term follow-up sessions, with one family cancelling and the other not attending. The waiting list was reduced by half.<br>**User impact:** Clinicians expressed high levels of satisfaction.<br>**Barriers and facilitators:** NR.	**Condition:**<br>Generic<br>**Population:**<br>Parents and children/young people<br>N=12<br>(only 10 attended appt)<br>**CYP Gender:**<br>Mixed<br>6m, 6f<br>**CYP Age:**<br>0-17yr<br>**Country:**<br>UK
**Wade (2022)**<br>**Aim**: Assess the feasibility (recruitment and retention) of an online 12-session guided self-help family-based treatment (GSH-FBT) for parents of CYP with AN.<br>**Design/methodology:** A feasibility study.<br>**Intervention:** 12 online modules accessed weekly by parents in which a clinician outlined FBT principles. Weekly Zoom support for up to 30 mins with a therapist (postgraduate clinical psychology trainees who had weekly supervision) helped parents apply the knowledge.	**Clinical outcomes:** CYP had an average weight gain of 6 kg (BMI centile effect size = 2.61, 95% CI: 1.77–3.44) and a decrease in eating disorder behaviours (effect size = 1.11, 95% CI: .27–1.95). Improvements in general mood and behaviours in the child, and the impact of eating disorder symptoms on their functioning. Parents reported improvements in knowledge, skills, and confidence in managing AN.<br>**Service efficiency:** NR.<br>**User impact:** The therapist's support was highly valued. The videos were generally found to be informative, but 5 families found the content not as helpful as they expected. From 187 eligible families, only 24 (13%) expressed interest and 16 of these completed the baseline measures, with 13 completing the WLI over a 6-month recruitment period. Adverse events were reported during WLI.<br>**Barriers and facilitators:** NR.	**Condition:**<br>Eating disorders (anorexia)<br>**Population:**<br>Parents<br>N=16<br>(67% of eligible participants)<br>**CYP Gender:**<br>NR<br>**CYP Age:**<br>13-15yrs<br>**Country:**<br>Australia
**White et al. (2022)**<br>**Aim:** To see if Dialectical Behaviour Therapy (DBT) is feasible and acceptable to parents. To compare DBT enhanced skills training with DBT adolescent format.<br>**Design/methodology:** natural, quasi-experimental design<br>**Intervention:** Weekly 60-minute multifamily session, where CYP and parents learn DBT skills together, weekly 60-minute skills strengthening and homework review session (parents and CYP	**Clinical outcomes:** NR.<br>**Service efficiency:** NR.<br>**User impact:** After completion of the WLI, half (50%) moved to further treatment, 18% had enough improvement that they left the service, 15% decided DBT was not the best treatment for them, 6% left for other reasons. Rates of completion of subsequent treatment were similar between the two groups (75% in those who had taken part in WLI vs 61% who remained on WL). Results indicate group format for DBT is acceptable for adolescents.<br>**Barriers and facilitators:** NR.	**Condition:**<br>Self-harm and suicide<br>**Population:**<br>N= 125 families<br>(48 (38.4%) chose WLI)<br>**CYP Gender:**<br>17 m, 108 f<br>**CYP Age:**<br>Mean 15.9 years (sd 1.11)<br>**Country:**<br>USA

Surname of 1st author, year and brief description of study	Evaluation of study	Conditions[a], N, Gender[b], Population[c], Age, Country
separate groups). 30-minute 1:1 check-in fortnightly.		

NOTES: [a]**Condition:** AN=anorexia nervosa, ASD=autism spectrum disorders, BEH=behavioural problems, CAMHS referral not specified, ED=eating disorder, GEN=generic, TGD=transgender; [b]**Gender:** Mixed[m]=mixed predominately birth-assigned male, Mixed[f]=mixed predominately birth-assigned female; [c]**Population:** Participants are defined as those who participated in the intervention. CYP=children and young people, P/C=parents/caregivers; **GSH-FBT:** guided self-help family-based treatment; **NR**=not reported
